# Management of infective endocarditis with cerebral complications

**DOI:** 10.1186/1749-8090-8-S1-O99

**Published:** 2013-09-11

**Authors:** W Fukuda, K Daitoku, M Minakawa, K Fukui, Y Suzuki, I Fukuda

**Affiliations:** 1 Department of Thoracic and Cardiovascular Surgery, Hirosaki University School of Medicine, Hirosaki, Japan

## Background

Management of patients with infective endocarditis complicated by neurological deficits is challenging. No clear management guidelines have been defined and the timing of surgery remains controversial. To encourage and guide cardiac surgeons, cardiologists and neurosurgeons to save patients with critically ill conditions, we have developed an algorithm for the management of IE patients. The purpose of this study is to evaluate our IE management algorithm.

## Methods

Thirty-eight adult patients with left-sided infective endocarditis undergoing valve surgery were analyzed. Before operation, enhanced brain CT was performed to rule out cerebral complication. Pre- and postoperative data were retrospectively reviewed to clarify whether our algorithm was effective. Sixteen patients having neurological complication (CVC group) were compared with 22 patients without neurological complication.

## Results

Age, sex, NYHA functional class, affected valve and pathogens were not different between two groups. Mean interval from onset of neurological dysfunction to cardiac operation was 27.8 ± 27.8 days (median 23 days). Of the 16 CVC group patients, 12 experienced cerebral infarction. Mass effects were seen in 3 patients, with 1 of these 3 patients died following aneurysm rupture. Mycotic aneurysm was detected in 4 patients, with 3 undergoing successful staged operations. Mortality and postoperative neurological exacerbation in CVC group was 6.3% (1 patient). Most patients who fulfilled the algorithm showed good outcomes.

## Conclusions

Our suggested management algorithm for infective endocarditis appears effective.

